# A quasi-experimental examination of how school-based physical activity changes impact secondary school student moderate- to vigorous- intensity physical activity over time in the COMPASS study

**DOI:** 10.1186/s12966-016-0411-9

**Published:** 2016-07-29

**Authors:** Stephen Hunter, Scott T. Leatherdale, Kate Storey, Valerie Carson

**Affiliations:** 1 Faculty of Physical Education and Recreation, University of Alberta, Edmonton, AB T6G 2H9 Canada; 2 School of Public Health and Health Systems, University of Waterloo, Waterloo, ON N2L 3G1 Canada; 3 School of Public Health, University of Alberta, Edmonton, AB T6G 2T4 Canada

**Keywords:** Adolescent, Youth, Environment, Longitudinal, Programs, Policy, Intervention

## Abstract

**Background:**

Adolescence is characterized by low moderate- to vigorous- intensity physical activity (MVPA) levels. Targeting the school setting can increase MVPA among a large proportion of adolescents. However, school-based physical activity interventions for adolescents remain largely ineffective. Therefore, the purpose of this study was to examine how naturally-occurring changes to school physical activity policy, recreational programming, public health resources, and the physical environment, impact adolescent MVPA over a 1-year period.

**Methods:**

Quasi-experimental longitudinal data from 18,777 grade 9–12 students (mean age = 15.1 ± 0.02 years), and 86 principals from 86 schools, participating in year 2 (2013–2014) and year 3 (2014–2015) of the COMPASS study (Ontario and Alberta, Canada) was used. Total MVPA over the previous week was self-reported at both time points using the COMPASS Student Questionnaire and average daily MVPA was calculated. Changes to physical activity policies, recreational programming, public health resources, and the physical environment were self-reported by school principals. Changes to the number and condition of physical activity facilities were objectively measured during school audits using the COMPASS School Environment Application. Multi-level modeling was used to examine change in student MVPA between schools that made changes and schools that did not. Models were adjusted for several student and school level confounders.

**Results:**

Over the 1-year period, 61 of 86 schools made physical activity related changes. Of these, 9 significantly changed student MVPA. However, only 4 of 9 schools’ changes increased student MVPA, including opening the fitness centre at lunch (β = 17.2, 95 % CI: 2.6–31.7), starting an outdoor club (β = 17.8, 95 % CI:7.4–28.1), adding a bike rack (β–14.9, 95 % CI:0.7–29.1), and adding weightlifting and run/walk clubs, archery, figure skating, increased access to the sports field, and improved condition of the outdoor basketball court (β = 15.5, 95 % CI: 5.2–25.7).

**Conclusions:**

Changes such as adding or increasing access to facilities, and adding multiple recreational programs, seemed to be effective for increasing student MVPA over the 1-year period. However, given the specificity of results, a one-size fits all approach may not be effective for increasing MVPA. Instead, school principals need to consider the resources within and surrounding their school, and the interests of the students.

## Background

Regular moderate- to vigorous-intensity physical activity (MVPA) is associated with several physical, mental health, and cognitive benefits in school-aged children and youth [1–3]. According to recent global estimates, approximately 80 % of adolescents failed to meet the recommended amount of MVPA for optimal health benefits [4]. Further, evidence has suggested that there is an actual decline in the amount of time spent in MVPA during adolescence [5, 6]. Given the adverse health consequences associated with physical inactivity [4, 7], identifying effective strategies to increase MVPA and promote a healthy active lifestyle among adolescents is warranted.

Ample research has identified individual correlates that can be targeted to increase MVPA among adolescents [8]. However, some models suggest that factors outside of the individual also have potential to influence MVPA. For instance, ecological models often recognize that the development of health-enhancing behaviours, such as MVPA, involves interactions between the individual (e.g., self-efficacy, enjoyment, attitudes) [9] and the multiple contexts (e.g., home, school, community) in which they are situated [10, 11]. Since most adolescents spend approximately 25 h each week in school throughout the school year [12, 13], the school environment represents one important context for shaping MVPA [3, 14–16].

Examination of the school environment’s influence on student MVPA has been a growing body of research within the last five years [17]. According to a recent systematic review examining aspects of the whole-school environment, it was found that activity setting, perceived teacher support, and intramurals were consistently positively associated with student MVPA [17]. However, the majority of included studies were cross-sectional in nature, and the evidence from the limited longitudinal and experimental studies was mixed [17]. Similar mixed results were found in a separate review, stemming from heterogeneity in terms of frequency, duration, and intensity of school-based PA interventions [18]. While the authors of the review identified several PA intervention successes for increasing MVPA in younger children, it was concluded that PA interventions targeting adolescents were ineffective [18].

Given the combined evidence, one way to strengthen the current literature would be to examine longitudinal associations using quasi-experimental designs [19, 20]. Examination of naturally-occurring changes that take place in real-world settings over time could reduce some of the potential external validity issues that have been associated with controlled trials [21], and address issues of causality inherit with cross-sectional studies [22–24]. Furthermore, quasi-experimental designs allow for the simultaneous observation of multiple interventions that occur in diverse settings. Such observations can be useful for identifying the most effective interventions for increasing adolescent MVPA. Therefore, the purpose of this study is to examine how naturally-occurring changes to secondary school physical activity (PA) policies, recreational programming, public health resources, and the physical environment, impact secondary student MVPA over a one-year period.

## Methods

### Design

The cohort for obesity, marijuana use, physical activity, alcohol use, smoking, and sedentary behaviour (COMPASS) is an ongoing quasi-experimental study that collects annual data regarding multiple health behaviours from secondary school students in grades 9 to 12 (aged 13 to 18 years), and the schools they attend in Ontario and Alberta, Canada [25]. COMPASS follows a cyclical process where student and school data is collected and used to generate school health profiles. The school health profiles are given back to the schools with feedback on their students’ health status, as well as information and resources that can be used to target identified problem areas. Schools then have the option to make a change themselves or contact a COMPASS knowledge broker for assistance with targeting identified problem areas. For all changes that occur, the COMPASS research team evaluates their impact on student health outcomes to generate practice-based evidence. The current study used longitudinal student- and school-level data from Ontario and Alberta schools in year two (Y_2_: 2013–2014) and year three (Y_3_: 2014–2015) of the COMPASS study. Data was collected using the COMPASS Student Questionnaire (Cq) [26], the COMPASS School Policies and Practices Questionnaire (SPP) (based off of the Healthy School Planner tool; [27]), and the COMPASS School Environment Application (Co-SEA) [28]. A full description of the study methods is available in print [25] or online [29]. Ethical approval was obtained from the University of Waterloo Office of Research Ethics and University of Alberta Research Ethics Board. All school boards and schools approved study procedures. Active-information passive-consent was sought from parents, and assent was obtained from participants on the data collection date. Parents or students could decline to participate at any time.

### Procedures

Each year students completed the Cq during class time, school principals filled out the SPP, and a COMPASS staff member performed an audit of the physical environment using the Co-SEA. The Cq collects individual student data on health behaviours, including MVPA, and demographic characteristics [25]. The SPP is a shorter, modified version of the previously validated Healthy School Planner tool [27] and captures information on school policies, programs, resources, and the environment related to student health [25]. The Co-SEA is a software application that can be downloaded to most mobile devices (e.g., cellular phone) and allows for pictures to be taken, stored, and assigned rankings. The Co-SEA was used by a COMPASS staff member to take pictures of PA facilities present within the school [28].

### Participants

In Y_2_, data from 79 Ontario and 10 Alberta schools was collected. A total of 57,229 students were enrolled in the 89 secondary schools with 79.15 % (*n* = 45,298) of eligible students completing the Cq. In Y_3_, data was collected from 78 Ontario and 9 Alberta schools. A total of 53,846 students were enrolled in the 87 secondary schools with 78.66 % (*n* = 42,355) of eligible students having completed the Cq. Missing respondents due to parental refusal accounted for 1.2 %, and 0.78 %, of the eligible sample in Y_2_, and Y_3_, respectively. The remainder of missing respondents were due to absenteeism or students being on a spare (i.e., unscheduled class) during the data collection, or student refusal. Furthermore, three schools dropped out from Y_2_ to Y_3_ due to administration changes and questionnaire length. Though one school was added in Y_3_ resulting in a sample of 87 secondary schools, this school was not included in the present study because it did not have any Y_2_ data. Therefore, 86 schools with complete data were included in this study.

To explore longitudinal changes among respondents, we paired Y_2_ and Y_3_ student-level data within schools, creating a longitudinal sample of 19,854 students from 86 schools. The paired sample accounted for 35.2 % of eligible Y_2_ respondents (*n* = 56,356), and 37.1 % of eligible Y_3_ respondents (*n* = 53,426). As expected, the 10,233 grade 12 students in Y_2_ that graduated were not in school in Y_3_, and the 11,070 grade 9 students that were newly admitted to participating schools in Y_3_ were not paired and were excluded. Other reasons for non-paired data included students who transferred schools, students who were had spare classes or were absent during the time of Y_2_ or Y_3_ data collection in their school, early school leavers, or inaccurate data provided in the data pairing measures on the Cq. Methodological details on the COMPASS data pairing procedures are available [30].

### Exposures

#### Subjective school-level changes

Changes to PA policies, recreational programming, use of public health units (i.e., a government health agency that carries out community health programs), and environment/equipment were assessed via single items on the SPP. Principals were provided with their previous year’s responses and were asked to report if any changes had occurred since the previous school year. If changes were made, they were then prompted to provide additional details about the change.

#### Objective school-level changes

Changes to quantity and condition of PA facilities were measured using the Co-SEA. Quantity of school PA facilities were recorded in both years by COMPASS research staff performing a school audit. Quantity changes were determined by subtracting the number of facilities present in Y_2_ from the number of facilities present in Y_3_. Condition of the PA facilities were measured each year on a 3-point scale (1 = ‘poor’, 2 = ‘adequate’, 3 = ‘good’). Condition changes were determined by subtracting conditions scores in Y_2_ from condition scores in Y_3_.

### Outcome

#### Change in student self-reported MVPA

MVPA was measured by two questions on the Cq. Students were required to complete the following item “Mark how many minutes of vigorous physical activity you did on each of the last 7 days. This includes physical activity during physical education class, lunch, after school, evenings, and spare time.” The same item was used to measure moderate physical activity, but instead of “vigorous,” the term “moderate” was substituted into the sentence. Responses were recorded in hours (0–4) and minutes (0, 15, 30, 45) for each day of the week. To help students better understand the questions they were given examples of vigorous (i.e., jogging, team sports, fast dancing, jump-rope, and any other physical activities that make you breathe hard and sweat) and moderate (i.e., lower intensity activities such as walking, biking to school, and recreational swimming) physical activities. Responses to these questions were added and averaged over the seven days to calculate daily MVPA. These items have demonstrated moderate test-retest reliability (ICC = 0.75); and slight criterion validity for MVPA (ICC = 0.25) against accelerometers [31], which is comparable to other self-reported measures used with adolescents [32–37].

### Covariates

#### Student-level covariates

Age, sex, ethnicity, weekly spending money, and physical education enrollment were considered covariates based on previous research examining their influence on MVPA [8, 38–40]. In addition, typical MVPA was also considered as a covariate, given its potential impact on MVPA. These variables were measured via single items on the Cq. There were seven response options for ethnicity. Based on frequency distributions, ethnicity was collapsed into two groups (“White,” and “non-White”). There were eight response options for weekly spending money ranging from “zero” to “$100+”, and “I do not know how much money I get each week.” To maximize sample size, participants who reported “I do not know” or who had missing data for this item were collapsed into one group [41]. There were three response options for physical education enrollment: (1) “Yes, I am taking one this term;” (2) “Yes, I will be taking one or have taken one this school year, but not this term;” and (3) “No, I am not taking a physical education class at school this year.” Students who reported different statuses from Y_2_ to Y_3_ formed one group, and students who reported the same status for each year formed the referent group. There were three response options for typical MVPA: (1) “Yes,” (2) “No, I was more active in the last 7 days;” and (3) “No, I was less active in the last 7 days.” Students who reported different responses in Y_2_ and Y_3_ formed one group, and students whose response was the same in each year formed the referent group.

#### School-level covariates

School size, school area level socioeconomic status, and school location were considered covariates based on previous research examining their influences on school PA facilities and program offerings [23, 24, 42]. School size was determined via school enrolment records and was entered into the model as a continuous variable. School area level socioeconomic status was constructed using the median household income of census divisions that corresponded with school postal codes, and was collected from 2011 National Household Survey data. School location was determined via school postal code, and Statistics Canada classifications were used to classify schools as “rural,” “small urban,” “medium urban,” and “large urban.” Based on frequency distributions, “rural” and “small urban” were collapsed to form one group, and “medium urban” and “large urban” were collapsed to form another group [43].

### Analysis

Analyses were completed using SAS version 9.4 (SAS Institute Inc., Cary, NC). Descriptive statistics were calculated for student-level and school-level variables using linear and logistic regressions that accounted for the clustering effect of schools. Likewise, the same procedures were used to compare demographic characteristics between included and excluded participants. To address the main purpose a three level, multi-level growth model was conducted using the MIXED procedure. Data was transposed from wide to long format so that time was nested in students, and students were nested in schools, with random intercepts included for students and schools. Consistent with other quasi-experimental research that looked at change over time [44], *each* school that made a PA related change between Y_2_ and Y_3_ was treated as a change group, while schools that made no PA related change between Y_2_ and Y_3_ were collapsed into one control group and served as the reference group. The multi-level growth model included time (Year), dummy variables for each change group compared to the reference group, and all student-level and school-level covariates. Additionally, to compare the impact of each change group compared to the reference group on change in student-level MVPA, a time*change interaction term was included in the model for each change group. Statistical significance was set a priori at *p* < 0.05.

## Results

Out of 19,854 students with paired data, students with missing variables were excluded (*n* = 808), and consistent with previous research students with an extreme MVPA change value (±3 SD) were removed (*n* = 269) [45], resulting in a final sample of 18,777 students. The included sample comprised of significantly older participants (Mean age = 15.07 years versus Mean age =15.01 years), more white participants (73.7 % versus 66.6 %); more female participants, (53.9 % versus 42.71 %), more participants whose typical week of MVPA status remained the same (58.0 % versus 48.5 %), and more participants whose physical education enrolment status remained the same (45.5 % versus 35.6 %) compared to the excluded group. Student and school demographic characteristics are listed in Tables 1 and 2, respectively. In Y_2_ mean MVPA for females and males was 107.87 min/day and 132.9 min/day, respectively. In Y_3_ mean MVPA for females and males was 100.6 min/day and 129.53 min/day, respectively. Overall, MVPA declined by 4.86 min/day.

**Table 1:** Characteristics of participants enrolled in year 2 (2013–2014) and year 3 (2014–2015) of the COMPASS study

Variable	Total (*n* = 18,777)
Mean Baseline Age (years)	15.1 (0.02)
Sex (%)	
Male	46.4 %
Female	53.6 %
Grade	
9	37.6 %
10	33.9 %
11	26.4 %
12	2.1 %
Ethnicity (% White)	73.7 %
Spending Money (weekly)	
I don’t know, NS	13.5 %
Zero	18.4 %
$1-$5	7.9 %
$6-$10	9.6 %
$11–20	16.7 %
$21–40	12.5 %
$41–100	11.8 %
$100+	9.7 %
PE enrollment	
Change in PE status from Y_2_ – Y_3_	54.5 %
Typical MVPA in previous week	
Change in Typical MVPA status from Y_2_ – Y_3_	41.9 %
MVPA	
Average Time 1 MVPA (min/day)	119.5 (1.4)
Average Time 2 MVPA (min/day)	114.1 (1.5)

*Note*: Continuous variables were expressed as a mean (standard error) and categorical variables were expressed as a percentage

*NS* not stated, *PE* physical education

**Table 2:** Characteristics of schools enrolled in year 2 (2013–2014) and year 3 (2014–2015) of the COMPASS study

Variable	Total (*n* = 86)
School Size	
Small (1–500)	37.2 %
Medium (501–1000)	51.1 %
Large (1000+)	11.6 %
School Location	
Rural	3.4 %
Small Urban	45.3 %
Medium Urban	15.1 %
Large Urban	36.0 %
School level SES	
$25000 – 50000	8.1 %
$50001–75000	68.6 %
$75001–10000	19.7 %
>$100000	3.4 %

*Note*: Categorical variables were expressed as a percentage

Of the 86 schools included in this study, 61 made PA related changes to at least one feature of the school environment between Y_2_ and Y_3_. Detailed descriptions of school changes are presented in Table 3. Briefly, none of the schools made any policy related changes, 15 schools made changes to recreational programming, two schools made changes to their use of public health units, two schools made changes to the subjective environment/equipment, and two schools made changes to both recreational programming and the subjective environment/equipment. Furthermore, 21 schools made changes to the physical environment within their school. Of these 21 schools, quantity changes occurred in five schools, condition changes occurred in 10 schools, and both quantity and condition changes occurred in six schools. Lastly, 19 schools reported multiple changes that encompassed combinations of changes to recreational programming, use of public health units, the subjective environment/equipment (as reported in SPP), and the physical environment (measured by Co-SEA).

**Table 3:** Description of physical activity related changes implemented between year 2 (2013–2014) and year 3 (2014–2015) of the COMPASS study

Description of Intervention	
Recreational Programming	
School 1	**SPP: Recreational Programming:** − *We have an active [special skills] program focused on Sports and Health. We have had concussion seminars for staff and then students. [Special skills] Sports students have leadership opportunities to lead sports related activities with our feeder schools.*
School 2	**SPP: Recreational Programming:** − *Right to Play, Play Academy. Leaders from the school mentoring elementary students on the role of physical activity. True Sport movement. Archery club added, mountain biking available.*
School 3	**SPP: Recreational Programming:** − *We are in the process of implementing an archery program which we hope will engage students not typically engaged in other physical activities. The most significant change is the establishment and implementation of our Health Champions committee in collaboration with [provincial health services]. This committee is composed of several staff members and has taken on a number of initiatives. The Health Champions organized a school-wide Health Fair during which there were a number of sessions offered to students ranging from hand-washing to archery to managing anxiety. Several community agencies and people were involved in the Fair as presenters. We hope to make this an annual event. Our Health Champions are also promoting healthy choices in the school and lobbying for things such as a bottle filling station. The Health Champions have also organized a number of lunch-time activities for students.*
School 4	**SPP: Recreational Programming:** − *This year, the school leadership class facilitated intramural activities during lunches - dodgeball, Tchuk-ball, dance-off, ping-pong, basketball, floor hockey.*
School 5 (+)	**SPP: Recreational Programming:** − *Fitness centre open at lunch as well.*
School 6	**SPP: Recreational Programming:** − *We have 2 full time athletic therapists who help athletes. Have a weight room and strength training coach. Conditioning and strength training available.*
School 7	**SPP: Recreational Programming:** − *Volleyball, badminton, yoga, and intramurals. At school we offer many opportunities for extra-curricular activities; YMCA teen night is free, basketball court is widely used at our school (outdoor). The school offered non-competitive sports clubs such as volleyball, badminton and basketball. Football was added and track. Tennis was offered, but did not run.*
School 8	**SPP: Recreational Programming:** − *[Physical education] classes hire outside instructors (yoga, Zumba, self-defence, etc.) and go to fitness clubs for specialty classes - The [health education] teacher brought in guest speakers as well; non-traditional/individual sports – golf, tennis, etc. Before/afterschool/during lunch students can play inside or outside – floor hockey, basketball, ping pong etc. We fundraise with fitness classes – Zumba. Use outdoor ed to take all grade 9 and “at risk” students Tree Top Trekking. Students adapt sports and instruct students with special needs for an afternoon.*
School 9	**SPP: Recreational Programming:** − *Offering archery club.*
School 10 (+)	**SPP: Recreational Programming:** − *An [outdoor] club was started as a result of a focus on health and wellness from student council – they have been involved in monthly hikes/outings.*
School 11 (−)	**SPP: Recreational Programming:** −*Terry Fox Run again this year.*
School 12	**SPP: Recreational Programming:** − *During warm weather connection classes are encouraged to be physically active. Intramural programs are underway.*
School 13	**SPP: Recreational Programming:** − *Encourage [school fitness] activities (6 week period).*
School 14	**SPP: Recreational Programming:** − *Christmas dance had to be cancelled due to lack of ticket sales. Addition of archery.*
School 15	**SPP: Recreational Programming:** − *Almost all the same but as a school this year we did not participate in the Terry Fox Run or Jump Rope for Heart.*
Role of Public Health	
School 16	**SPP: Public Health:** − *Working with [public health unit] to pilot some projects [physical education intervention].*
School 17	**SPP: Public Health:** − *This year the grade 9 [physical and health education] students are involved in an intervention study with the [university].*
Subjective Measurement of Environment/Equipment Changes	
School 18	**SPP: Environment/Equipment:** − *more opportunities for exercise and increased activity equipment for ALL students.*
School 19	**SPP: Environment/Equipment:**− *Showers now are individual with curtains for more privacy.*
Multiple Changes measured by SPP	
School 20	**SPP: Recreational Programming:** − *Offering after school program for students from remote communities. Partnership with [non-profit organization focussed on providing physical activity opportunities for disadvantaged youth] - Looking to install basketball nets for students to use outside. Expansion of non-competitive options like yoga and Crossfit. Classes take kids out for a walk. Equipment available for kids at lunch e.g., balls, hockey sticks.* **SPP: Environment/Equipment:** − *Washrooms renovated.*
School 21	**SPP: Recreational Programming:** − *Intramurals offered at lunch: Well attended - October: [charity run].* **SPP: Environment/Equipment:** − *Yes a change - No secure lockers, could change in private in the washrooms.*
Objective Measurement of Environmental Changes measured by Co-SEA	
Quantity	
School 22 (−)	**Co-SEA:** Added a dance studio.
School 23 (+)	**Co-SEA:** Added a bike rack.
School 24	**Co-SEA:** Added a bike rack.
School 25	**Co-SEA:** Added a bike rack.
School 26	**Co-SEA:** Added a bike rack, tennis court, and outdoor basketball court. Removed the fitness/weight room.
Condition	
School 27	**Co-SEA:** Condition of the tennis court improved.
School 28	**Co-SEA:** Condition of the outdoor track improved.
School 29	**Co-SEA:** Condition of the gym improved.
School 30	**Co-SEA:** Condition of the fitness/weight room improved.
School 31 (−)	**Co-SEA:** Condition of the fitness/weight room improved.
School 32	**Co-SEA:** Condition of the fitness/weight room improved, and condition of the gym worsened.
School 33	**Co-SEA:** Condition of the field worsened.
School 34	**Co-SEA:** Condition of the outdoor track worsened.
School 35	**Co-SEA:** Condition of the fitness/weight room worsened.
School 36	**Co-SEA:** Condition of the fitness/weight room worsened.
Quantity & Condition	
School 37	**Co-SEA:** Added 2 fitness/weight rooms, and the condition of gym improved.
School 38	**Co-SEA:** Added baseball diamond, removed outdoor basketball court, and the condition of the fitness/weight room improved.
School 39	**Co-SEA:** Added dance studio, and the condition of the indoor facility [not specified] improved.
School 40	**Co-SEA:** Added fitness/weight room, removed yoga room, and the condition of fitness/weight room improved.
School 41	**Co-SEA:** Added two fitness/weight rooms and the condition of outdoor basketball court improved.
School 42	**Co-SEA:** Added field, and the condition of the field, and the outdoor track worsened.
Multiple School Changes (Measured by Co-SEA & SPP)	
School 43	**Co-SEA:** Added other outdoor facility (crossfit, foobtall equipment), removed a bike rack, and the condition of the field worsened. **SPP: Recreational Programming:**− *Hockey Canada skills academy a 2 credit physical education program. 5 days out of 10 on ice at a recreation center.* **SPP: Environment/Equipment:**− *Yes, no bike racks due to school renovations.*
School 44	**Co-SEA:** Added an outdoor basketball court, added a closed road for hockey, biking; and the condition of fitness/weight room improved. **SPP: Public Health: **− *Walking program became defunct over the past year, we are examining ways to get it started up again as well as implementing a house system pedometer walking challenge as recommended in 13/14 COMPASS results.* **SPP: Recreational Programming: **− *“house” system has been implemented, gr. 9–12 students now have additional opportunities to participate in friendly grade by grade sports competitions on a monthly basis Grade 7/8 students practiced after school and participated in [community kids marathon].* **SPP: Environment/Equipment:** − *Major renovation in the school allowed the creation of a cross-fit space, being well utilized by phys. ed department and as part of the after school fitness program.*
School 45	**Co-SEA:** Added a long jump pit. **SPP: Recreational Programming:** − *Students have access to basketballs during non-instructional times.*
School 46	**Co-SEA:** Added an outdoor volleyball court. **SPP: Recreational Programming:** − *No [health] walks.*
School 47	**Co-SEA:** Condition of the gym, and fitness/weight room worsened. **SPP: Recreational Programming:** − *[Jane] did less this year because she was so busy.* **SPP: Environment/Equipment:** − *Newer curtains were added to shower stalls in girls’ change room.*
School 48	**Co-SEA:** Added a fitness/weight room and two fields. **SPP: Recreational Programming:** − *No intramurals.*
School 49 (+)	**Co-SEA:** Condition of the outdoor basketball court improved. **SPP: Recreational Programming:** − *All students can join the weight lifting club or the 100 km walk/run club. Added archery and figure skating.* **SPP: Environment/Equipment:** − *Students have access to the sports field at lunch in there is no physical education class using it.*
School 50	**Co-SEA:** Added a yoga room. **SPP: Recreational Programming:** − *School uses a program called Kids Sport to help fund underprivileged students who cannot afford to be a part of school programs.*
School 51	**Co-SEA:** Added an outdoor basketball court, and condition of the outdoor track improved. **SPP: Recreational Programming: **− *No longer host relay for life -Shine On program has been added for self-esteem/self-awareness. Yoga and nutrition for female students 1x week.* **SPP: Environment/ Equipment:** *New rubberized track and outdoor basketball court.*
School 52 (−)	**Co-SEA:** Condition of the fitness/weight room improved. **SPP: Recreational Programming:** − *[Received fitness grant] from the Ministry of Education - Built fitness 101 room with spin bikes, bose balls, ping pong, and shuffleboard. Also provided 10 sessions each after school for spinning, yoga, Zumba. Gym open every lunch for student use.*
School 53	**SPP: Recreational Programming:** − *Wellness Week - One week of Wellness Week 2015 focused on Play or physical activity. A Wii Dance-off competition against another secondary school was held with 175 students and staff dancing together for 20 min. Terry Fox Run, Inside Ride, intramural programming, Semi Pro Basketball league - only open to students who didn't play on a HC team. Dodge ball competition, flag football league, varsity sports programs, and weight room memberships. Membership for students and staff in a fully equipped weight room; money raised from memberships used to purchase equipment.* **SPP: Public Health:** − *Involvement in Wellness Week.* **SPP: Environment/Equipment:** − *Construction of an outdoor basketball court for students and community members to use.*
School 54	**SPP: Recreational Programming:** − *Partnership with Recreation Centre to allow free access to weight room. Archery club added -Outdoor Education instead of just canoe activities -Unsure if the school participated in the Terry Fox Run.* **SPP: Environment/Equipment:** − *Girls’ showers all have private stalls and curtains -Students also access physical activity facilities at [another school].*
School 55	**Co-SEA:** Added a yoga room. **SPP: Recreational Programming:** − *Supervised gym time during lunch hours, hopefully supervised fitness room during lunch hours, and supervised fitness room after school hours.* **SPP: Environment/Equipment:** − *Curtains in female shower stalls.*
School 56	**Co-SEA:** Added a fitness/weight room. **SPP: Recreational Programming:** − *Continued building relationships with community partners (i.e., with senior league golf and the curling club). Hockey academy (a 2 credit physical education package) is now available and is a focused course emphasizing specific activity skill development and conditioning. Soccer academy will be available next year. Healthy active living education courses are now 14 sections. Intramural expansion continues. Badminton club is growing.*
School 57	**Co-SEA:** Removed 2 fitness/weight rooms, and condition of the field improved. **SPP: Recreational Programming:** − *Boarding students have increased access to use fitness studio (now can use without adult supervision) if they go with a buddy.*
School 58 (−)	**Co-SEA:** Condition of the field improved. **SPP: Recreational Programming:** −*added dance club, athletic council to sports selection.*
School 59	**Co-SEA:** Condition of the field worsened **SPP: Recreational Programming:** − *Added outdoor education and a walking club. Ultimate Frisbee. Added a new girls’ only fitness club and there will be 2 sections running next year.*
School 60	**Co-SEA:** Added a yoga room, condition of the fitness/weight room improved. **SPP: Recreational Programming:** − *No [charity walk] this year.*
School 61	**Co-SEA:** Condition of the fitness/weight room worsened, and condition of the field worsened. **SPP: Recreational Programming:** − *The school has not offered intramurals so far this year (completed Nov/Dec 2014).*

*Note:* Italicized text represents qualitative response from school staff/principals, bold text indicates measurement tool. (+) indicates change resulted in significant increase in student MVPA, (−) indicates change resulted in significant decrease in student MVPA

As shown in Table 4, of the 61 schools that had PA related changes, a significant change in student MVPA was observed in nine schools. Of these nine schools, four schools’ changes resulted in a significant increase in student MVPA, while significant decreases in student MVPA occurred in five schools. Significant increases in MVPA were observed in School 5, 10, 23, and 49. School 5 had their fitness centre open at lunch (β = 17.1765, 95 % CI: 2.6079 to 31.7451). School 10 started an *out and abouters* club as a result of a focus on health and wellness from the student council, which involved monthly hikes and outings (β = 17.7959, 95 % CI: 7.4354 to 28.1564). School 23 added a bike rack (β = 14.919, 95 % CI: 0.6891 to 29.1488). Lastly, School 49 improved the condition of the outdoor basketball court, provided students with opportunities to join the weight lifting club or the 100 km walk/run club, added archery and figure skating, and enabled access to the sports field at lunch if it was not already occupied by the PE class (β = 15.4671, 95 % CI: 5.2029 to 25.7312).

**Table 4:** Multilevel modeling examining the impact of school physical activity related changes on student self-reported MVPA between year 2 (2013–2014) and year 3 (2014–2015) of the COMPASS study

Parameter	β	95 % CI		Standard error	T value	*P* value
		lower	upper			
Intervention* Year						
Control Schools (*n* = 25)	**Ref**	−	−	−	−	−
School 1	−1.4126	−20.8311	18.0059	9.907	−0.14	0.8866
School 2	−3.3077	−11.159	4.5435	4.0056	−0.83	0.4089
School 3	−4.6045	−19.8508	10.6419	7.7784	−0.59	0.5539
School 4	−3.5289	−11.6699	4.6122	4.1534	−0.85	0.3955
School 5	**17.1765**	**2.6079**	**31.7451**	**7.4326**	**2.31**	**0.0208**
School 6	−6.0454	−14.6058	2.5149	4.3673	−1.38	0.1663
School 7	−5.6419	−19.4357	8.1518	7.0373	−0.8	0.4227
School 8	−3.4567	−10.1445	3.2312	3.412	−1.01	0.311
School 9	1.0997	−10.2788	12.4782	5.8051	0.19	0.8497
School 10	**17.7959**	**7.4354**	**28.1564**	**5.2857**	**3.37**	**0.0008**
School 11	**−14.1243**	**−22.4178**	**−5.8309**	**4.2312**	**−3.34**	**0.0008**
School 12	−8.0511	−18.1531	2.0509	5.1538	−1.56	0.1183
School 13	−1.7797	−17.3599	13.8005	7.9487	−0.22	0.8228
School 14	8.0656	−8.8876	25.0187	8.6492	0.93	0.3511
School 15	−4.0273	−13.2666	5.212	4.7137	−0.85	0.3929
School 16	2.2906	−4.6181	9.1993	3.5247	0.65	0.5158
School 17	−0.6574	−12.0688	10.754	5.8219	−0.11	0.9101
School 18	15.3825	−1.4601	32.2251	8.5928	1.79	0.0734
School 19	2.3569	−8.0037	12.7174	5.2857	0.45	0.6557
School 20	−1.9814	−12.2218	8.2591	5.2245	−0.38	0.7045
School 21	4.6348	−5.9797	15.2493	5.4153	0.86	0.3921
School 22	**−8.994**	**−17.6915**	**−0.2965**	**4.4373**	**−2.03**	**0.0427**
School 23	**14.919**	**0.6891**	**29.1488**	**7.2598**	**2.06**	**0.0399**
School 24	0.9965	−10.0384	12.0314	5.6298	0.18	0.8595
School 25	−7.8715	−15.8157	0.07262	4.0529	−1.94	0.0521
School 26	−2.8055	−16.3117	10.7007	6.8906	−0.41	0.6839
School 27	2.4335	−4.6778	9.5449	3.6281	0.67	0.5024
School 28	−1.057	−11.159	9.045	5.1538	−0.21	0.8375
School 29	−1.7557	−12.5323	9.0209	5.498	−0.32	0.7495
School 30	5.2861	−4.8614	15.4336	5.1771	1.02	0.3072
School 31	**−11.0801**	**−21.2506**	**−0.9096**	**5.1888**	**−2.14**	**0.0327**
School 32	−11.3142	−26.8944	4.266	7.9487	−1.42	0.1546
School 33	1.5632	−10.6565	13.783	6.2343	0.25	0.802
School 34	−1.6234	−11.7938	8.5471	5.1888	−0.31	0.7544
School 35	−6.9064	−18.7374	4.9246	6.0359	−1.14	0.2525
School 36	−9.0054	−19.866	1.8552	5.5409	−1.63	0.1041
School 37	−0.2115	−8.7585	8.3356	4.3605	−0.05	0.9613
School 38	8.741	−12.6266	30.1085	10.9013	0.8	0.4227
School 39	7.437	−4.8238	19.6978	6.2552	1.19	0.2345
School 40	−6.0121	−15.6917	3.6675	4.9383	−1.22	0.2235
School 41	−2.1108	−14.9006	10.679	6.5251	−0.32	0.7463
School 42	−5.9664	−16.9423	5.0094	5.5997	−1.07	0.2867
School 43	0.7273	−6.5046	7.9592	3.6896	0.2	0.8437
School 44	−0.9814	−18.5225	16.5598	8.9492	−0.11	0.9127
School 45	8.9158	−6.4947	24.3263	7.8621	1.13	0.2568
School 46	5.7191	−3.9605	15.3987	4.9383	1.16	0.2468
School 47	5.416	−8.6849	19.517	7.194	0.75	0.4515
School 48	9.2006	−0.02176	18.4229	4.705	1.96	0.0505
School 49	**15.4671**	**5.2029**	**25.7312**	**5.2366**	**2.95**	**0.0031**
School 50	−5.675	−19.6505	8.3006	7.1301	−0.8	0.4261
School 51	−2.9709	−12.5342	6.5924	4.879	−0.61	0.5426
School 52	**−11.4782**	**−22.6037**	**−0.3528**	**5.676**	**−2.02**	**0.0432**
School 53	3.1128	−4.507	10.7326	3.8875	0.8	0.4233
School 54	20.6528	−0.2776	41.5831	10.6783	1.93	0.0531
School 55	4.5232	−6.1178	15.1642	5.4288	0.83	0.4048
School 56	2.3501	−15.3163	20.0164	9.013	0.26	0.7943
School 57	−6.0286	−15.0386	2.9814	4.5967	−1.31	0.1897
School 58	**−10.3547**	**−18.7093**	**−2.0001**	**4.2624**	**−2.43**	**0.0151**
School 59	11.0406	−0.3379	22.4191	5.8051	1.9	0.0572
School 60	0.03211	−7.9226	7.9868	4.0583	0.01	0.9937
School 61	−8.2886	−22.928	6.3508	7.4687	−1.11	0.2671

*Note*: Bolded values are significant (*p* < 0.05). Adjusted for age, sex, ethnicity, physical education enrolment, weekly spending money, typical PA, school size, school location, and school area-level SES

*Represents an interaction

Significant decreases in student MVPA were observed in School 11, 22, 31 52, 58. School 11 offered the Terry Fox Run (i.e., charity run; β = −14.1243, 95 % CI: −22.4178 to −5.8309). School 22 added a dance studio (β = −8.994, 95 % CI: −17.6915 to −0.2965). School 31 improved the condition of their fitness/weight room (β = −11.0801, 95 % CI: −21.2506 to −0.9096). School 52 improved the condition of their fitness/weight room, and received a grant from which they built an alternate fitness room with additional equipment, after school sessions, and had it open during lunch hour for student use (β = −11.4782, 95 % CI: −22.6037 to −0.3528). Lastly, School 58 improved the condition of their field, and added a dance club and athletic council (β = −10.3547, 95 % CI: −18.7093 to −2.001). No other interventions produced significant results.

## Discussion

The purpose of this study was to examine how naturally occurring changes to PA policy, recreational programming, public health resources, and the physical environment within schools impacted student MVPA over a one-year period. We found that changes to some aspects of recreational programming (e.g., PA-related clubs) and the physical environment (e.g., addition of bike rack, fitness room) were associated with a significant change in student MVPA. Changes to public health resources proved not to be significant. Further, no policy changes occurred in any participating schools between Y_2_ and Y_3_, suggesting an opportunity for more targeted action moving forward.

To our knowledge this is the first study that looked at how naturally occurring school PA-related changes impacted student MVPA over time. There were nine schools’ changes that resulted in significant student MVPA changes over the one-year period. Considering the current study looked at change over time, it is important to keep in mind that this was not an examination of the presence or absence of policies, recreational programming, public health resources, or features of the physical environment. Therefore, it is quite possible for the control schools (*n* = 25) to have had existing initiatives in place and did not feel the need to make any PA related changes. As a result, this could explain the mixed impacts on student MVPA from what seemed to be positive PA school-level changes. Given the paucity of longitudinal studies examining the impact of school-led changes over time, it is difficult to directly align these findings with evidence from the current literature. However, ecological models [11] and previously identified associations are available to help support and interpret the results of this study.

Changing only aspects of the physical environment resulted in increased MVPA in one school, and decreased MVPA in two schools. The addition of a bike rack by school 23 resulted in increased student MVPA and is of particular interest considering two other schools also made this change, while a third school incorporated it among other changes. However, the addition of a bike rack in these three other schools did not significantly impact student MVPA. In previous research, the presence of bike racks alone have not been associated with student MVPA [22–24]. However, there is evidence to suggest that bike riding is a popular activity among high school students [46], and that active transportation (e.g., biking to school) is one way for students to engage in MVPA [47]. While this study did account for school-level variability and covariates, it did not observe features of the neighbourhood built environment that may influence MVPA [48]. Therefore, differences within the surrounding community environments of these schools may explain why the addition of a bike rack significantly impacted student MVPA in only one out of four schools. Another physical environment change that significantly impacted MVPA was the addition of a dance studio, which resulted in a decrease in student MVPA. In previous research dance studios typically have not been significantly associated with student MVPA [22–24]; therefore, it is unclear why student MVPA decreased in school 22. Restructuring space for dance studios has been suggested as a potential way to increase MVPA among low active groups [49]. However, given the results from this study it appears that more research is needed to determine whether adding a dance studio is a viable solution for schools looking to increase student MVPA. Finally, the last physical environment change that impacted MVPA was improving the condition of the fitness/weight room in school 31, which resulted in decreased student MVPA. Previously, facility condition has been positively associated with MVPA in both males and female students [50]. In the current study a change to facility condition was reported in 10 schools, in which five were specific to the fitness/weight room, yet only one of these significantly impacted student MVPA. Therefore, changing the condition of PA facilities may not be an effective strategy for schools to improve student MVPA as it appears to have a null or negative impact. Given the combined results, it appears that more research is needed examining the impact of objectively measured changes to the physical environment on student MVPA.

Changes to only recreational programming resulted in significant student MVPA changes in three schools. Of these, increased MVPA changes occurred from adding an out-and-abouters club in school 10, and from increasing access to the fitness centre at lunch in school 5. Given that the presence of a room with cardio or weightlifting equipment has previously been associated with increased odds of being physically active during recess [51], it does not seem out of place that students attending school 5 had increased MVPA. Further, it seems intuitive that offering an outdoor club in school 10 stemming from a student leadership initiative would also have a positive influence on student MVPA. However, it is unclear why the addition of a charitable run (Terry Fox Run) in school 11 resulted in a decrease in student MVPA. Although there is little evidence to suggest that one-time events are effective for increasing MVPA [52, 53], it should be noted that it was unknown whether the Terry Fox Run occurred within the recalled week of MVPA (previous week). Therefore, it is possible that some other change not captured in the SPP or Co-SEA was responsible for the observed decrease in student MVPA in this school. Consequently, it appears that more research is needed to examine how one-time events held by schools impact student MVPA over time.

Changes to both the physical environment and recreational programming occurred in three schools. Intuitively, an increase in MVPA was observed in school 49 which added a weightlifting club, a 100 km run/walk club, archery and figure skating, increased access to the sports field at lunch, and improved condition of the outdoor basketball court. However, surprisingly decreased MVPA was observed in school 52 that had built a fitness room complete with spin bikes, bose balls, ping pong, and shuffle board, provided 10 sessions each of spin, yoga, and Zumba, and increased access to the gym at lunch. A decrease in MVPA was also observed in school 58, which added a dance club and improved the condition of their field. Previous research has found that alternate rooms for PA have been positively associated with MVPA [22–24]. Therefore, it is interesting that we observed decreased MVPA in school 52 for building a fitness room. One potential explanation could be the timing of data collection, as it was unclear how close the renovations occurred to the actual data collection date. Hence, it will be interesting to see how this change impacts student MVPA in the COMPASS year four data collection. Another potential reason for the difference in results observed between schools could be the extra-curricular activities that were offered. For example, increased MVPA occurred in school 49 from incorporating multiple new activities (e.g., weightlifting, run/walk club, archery, figure skating), which could have appealed to a broader range of male and female students and resulted in a larger proportion of students being active [46]. While there is evidence to suggest that activities offered in school 52, such as spin, yoga, and dance are some of the least preferred activities and may only be of interest to females [46, 54]. Lastly, the observed differences in results could be due to implementation success. Previous research has identified support from school principals, physical space, and scheduling with other school activities (e.g., varsity teams) as factors that can facilitate or impede the ability to offer extra-curricular activities [55, 56]. In addition, student hunger, after-school transportation, and other student commitments (e.g., jobs, tutoring, family) were identified in previous research as potential issues that could influence participation in extra-curricular activities [55, 56]. Given that the current study did not assess these factors, an examination of the facilitators and barriers experienced by these three schools that made changes to recreational programming could provide a better understanding of the results.

Schools often cited policies that were embedded at the provincial level such as the daily physical activity initiative, and the physical education curriculum. However, these are not school-level policies and therefore were not considered an appropriate exposure for this study. Previous research has found that policies implemented beyond the school-level have experienced implementation issues [57, 58], and that there seems to be a lack of communication between policy makers, school board officials, principals, and teachers [59]. Therefore, future research should continue to examine PA policies and evaluate the relationship between policies developed at the school level and student MVPA.

Piercy et al., 2015 [60] suggested that public health units can encourage schools to adopt health promoting programs and act as a facilitator between the school and local community resources. However, only 4/61 schools changed the way they collaborated with public health units, with none of these changes significantly impacting student MVPA. Of these four schools, three offered PA programs, and one was in the process of working with their public health unit. One potential reason for the lack of significant results stemming from these schools could be that these were new programs being implemented and it could take a while for them to run efficiently. Future research should continue to assess the relationship between schools and local public health units in order to identify the most effective strategies for increasing the amount of time students spend in MVPA.

While the results of this study are mixed, they solidify previous recommendations in which multiple school contexts such as the physical, social, and policy environments need to be examined concurrently in order to better understand how the complete school environment influences student MVPA [17]. Although this study assessed the physical, and policy environments, the social environment was not addressed. Understanding how previously associated factors such as perceived teacher support [61–63] and feelings of school connectedness [64, 65], could have aided in the interpretation of the results. Schools may have sufficient facilities and a variety of activities to offer, however students may still need to feel supported by school personnel or have a sense of school connectedness before they engage in the opportunities that are provided. Therefore, more research is needed in order to understand how feelings of school connectedness, and teacher support are associated with the amount of time students spend in MVPA.

Strengths of this study included the quasi-experimental design, longitudinal data, objective and subjective measures of the physical environment, and the use of active-information passive-consent parental protocol. The COMPASS study uses active-information passive-consent parental protocol for its capability to reduce school-level variance estimates, increase participation rates to obtain a representative sample of the entire student population within a school, reduce the risk for obtaining a biased sample, obtain accurate information regarding substance use, and ensure student confidentiality [66]. Despite these strengths mentioned, there were limitations that need to be considered. The COMPASS study purposely sampled school boards that met a predetermined inclusion criterion and therefore is not a representative sample of all Ontario and Alberta schools. As a result, this could limit the generalizability to larger, English speaking schools [25, 67]. Another limitation was the use of a self-reported MVPA, which often results in overestimation of MVPA in adolescents compared to objective measures, such as accelerometers [68]. Further self-report measures are not as accurate in determining different intensities of PA as objective measures. However, given feasibility issues such as cost and time that are associated with objective measurement (e.g., accelerometers), self-reported measures like the Cq are acceptable for use in large samples. Further, if over-reporting did occur, it likely occurred at both times, minimizing the impact on MVPA change over time. Lastly, there are potential limitations regarding the use of the Co-SEA. Although the Co-SEA allows for objective measurement of the quantity of facilities present in physical environment, data collectors are required to subjectively assess the condition of these facilities. While training was provided to ensure a high degree of reliability was achieved, measurement error may still have occurred.

## Conclusion

This study provided a quasi-experimental observation of how naturally-occurring school changes to PA policy, recreational programming, public health resources, and the physical environment impacted student MVPA over time. Based on the changes’ that resulted in increased MVPA, it appears that providing increased access and multiple opportunities to be active may be an effective strategy for increasing MVPA in secondary school students. However, it was also found that the same school-level changes had different impacts on student MVPA. Further, in some schools even intuitively positive changes negatively impacted student MVPA. From an ecological perspective, it is possible that sources of influence at the interpersonal or community level could be interacting with these school-level changes [11]. Considering the dynamic nature of the school and its components (e.g., staff, students, surrounding community), future observations should assess multiple levels of the ecological model. This would be beneficial for understanding how these school components interact with one another, and the surrounding community to influence the amount of time secondary students spend in MVPA. Given the specificity of these results, it may be important for school principals to consider both the internal (e.g., staff, facilities) and external (e.g., community features) resources they have, as well the interests of the students in order to develop and deliver effective strategies for increasing student MVPA.

## Abbreviations

MVPA, moderate-to vigorous-intensity physical activity; PA, physical activity
